# Data on the effect of target temperature management at 32–34 °C in cardiac arrest patients considering assessment by regional cerebral oxygen saturation: A multicenter retrospective cohort study

**DOI:** 10.1016/j.dib.2018.02.050

**Published:** 2018-02-24

**Authors:** Yuka Nakatani, Takeo Nakayama, Kei Nishiyama, Yoshimitsu Takahashi

**Affiliations:** a Department of Health Informatics, Kyoto University School of Public Health, Yoshidakonoecho, Sakyo-ku, Kyoto City, Japan; b National Hospital Organization Kyoto Medical Center, Fukakusa-mukaihatakecho, Fushimi-ku, Kyoto City, Japan

## Abstract

This data article contains raw data and supplementary analyzed data regarding to the article entitled “Effect of target temperature management at 32–34 °C in cardiac arrest patients considering assessment by regional cerebral oxygen saturation: A multicenter retrospective cohort study”. We examined the effectiveness of target temperature management (TTM) at 32–34 °C considering degrees of patients’ cerebral injury and cerebral circulation assessed by regional cerebral oxygen saturation (rSO_2_). The research is a secondary analysis of prospectively collected registry, in which comatose patients who were transferred to 15 hospitals in Japan after out-of-hospital cardiac arrest (OHCA), and we included 431 study patients. Propensity score analysis revealed that TTM at 32–34 °C decreased all-cause mortality in patients with rSO_2_ 41–60%, and increased favorable neurological outcomes in patients with rSO_2_ 41–60% in the original research article. With regard to the balance of covariates of propensity-score matching (PSM) and inverse-probability weighting (IPW) analyses, some covariates were not well balanced after the analyses between groups. The overlap plots indicate the overlap of densities of the propensity scores are low in group rSO_2_ 41–60% and group rSO_2_ ≥ 61%. When patients were limited to those who achieved return of spontaneous circulation (ROSC) until/on hospitals arrival, TTM still tended to decrease all-cause mortality and increase favorable outcomes in group rSO_2_ 41–60%.

**Specifications table**

**Table:** 

Subject area	Medical science
More specific subject area	Post resuscitation care
Type of data	Tables, figures
How data was acquired	Survey
Data format	Raw data, statistically analyzed data
Experimental factors	Does not apply
Experimental features	The treatment, target temperature management (TTM) with 32–34 °C (12–24 h) was conducted by the discretion of the attending physician.
Data source location	Japan
Data accessibility	Data is available in this article
Related research article	Effect of target temperature management at 32–34 °C in cardiac arrest patients considering assessment by regional cerebral oxygen saturation: A multicenter retrospective cohort study *(in press)*

**Value of the data**

• The data contain raw data and supplementary contents of our original paper, and these are important information for interpretation the results of original research.
• TTM at 32–34 °C could be still effective when patients with rSO_2_ 41–60% were limited to who achieved ROSC until/on hospital arrival, excluding patients achieved ROSC after hospital arrival.
• The covariates of PSM and IPW analysis were not well balanced, and the overlap plots indicate the overlap of densities of the propensity scores are low in group rSO_2_ 41–60% and group rSO_2_ ≥ 61%.
• The use of TTM at 32–34 °C could be effective in patients with specific degrees of cerebral injury, but the result should be interpreted carefully.

## 1 Data

**Fig. 1:**
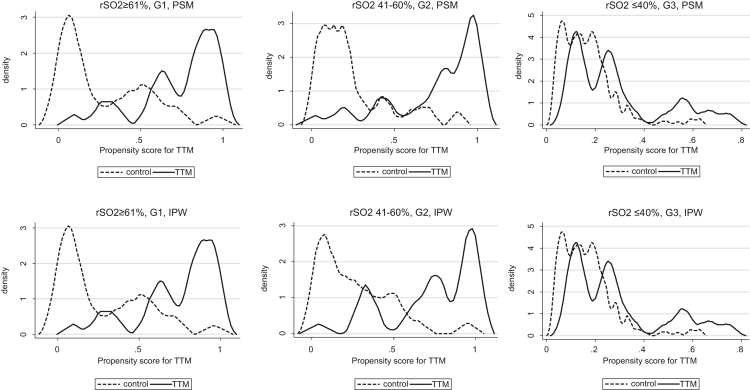
Overlap plots of propensity score matching analysis and inverse probability of treatment weighting for all-cause mortality.

**Fig. 2:**
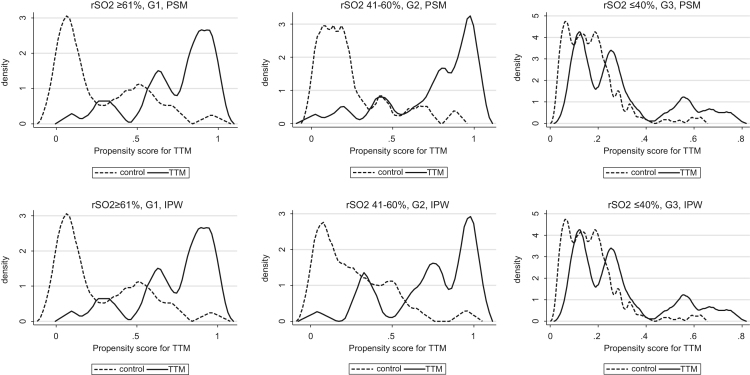
Overlap plots of propensity score matching analysis and inverse probability of treatment weighting for favorable neurological outcomes.

We examined the effectiveness of TTM at 32–34 °C considering degrees of patients’ cerebral injury and cerebral circulation assessed by regional cerebral oxygen saturation (rSO_2_). This is a secondary analysis of prospectively collected registry [1], [2], in which comatose patients who were transferred to 15 hospitals in Japan after out-of-hospital cardiac arrest (OHCA), and we included 431 study patients (Table S1) [3]. In original research article, propensity score analysis revealed that TTM at 32–34 °C decreased all-cause mortality in patients with rSO_2_ 41–60% (average treatment effect on the treated [ATT] by propensity score matching [PSM] −0.51, 95%CI −0.70 to −0.33; ATT by inverse probability of treatment weighting [IPW] −0.52, 95%CI −0.71 to −0.34), and increased favorable neurological outcomes in patients with rSO_2_ 41–60% (ATT by PSM 0.50, 95%CI 0.32–0.68; ATT by IPW 0.52, 95%CI 0.35–0.69). TTM at 32–34 °C could be effective to decrease all-cause mortality in comatose OHCA patients with rSO_2_ 41–60% on hospital arrival. Table 1, Table 2, Table 3, Table 4 show that the covariates of PSM and IPW analysis were not well balanced. The overlap plots (Fig. 1, Fig. 2) show the overlap of densities of the propensity scores are low in group rSO_2_ 41–60% and group rSO_2_ ≥ 61%, this indicates the overlap assumption on the treatment effect on the potential-outcome models may be violated. Table 5 shows that TTM could be still effective when patients with rSO_2_ 41–60% were limited to who achieved ROSC until/on hospital arrival, excluding patients achieved ROSC after hospital arrival.

**Table 1:** Balance of covariates of propensity score matching analysis for all-cause mortality^a^.

**Covariates**	**rSO**_**2**_**≥ 61%, G1 (*N* = 68, 34 pairs)**				**rSO**_**2**_**41–60%, G2 (*N* = 67, 31 pairs)**				**rSO**_**2**_**15–40%, G3 (N=296, 54 pairs)**			
	**SD**		**Variance ratio**		**SD**		**Variance ratio**		**SD**		**Variance ratio**	
	**Before matching**	**After matching**	**Before matching**	**After matching**	**Before matching**	**After matching**	**Before matching**	**After matching**	**Before matching**	**After matching**	**Before matching**	**After matching**
Sex	0.36	−0.37	0.65	2.63	0.25	1.09	0.82	1.0	0.28	0.040	0.86	0.97
Age	0.38	−0.14	0.83	2.25	−1.52	0.11	2.47	0.99	−0.18	−0.22	0.69	0.77
Location of cardiac arrest	0.37	−0.16	1.04	1.66	0.71	−0.40	1.59	0.78	0.41	−0.074	1.23	0.97
Witness	0.092	−0.58	0.84	–	0.13	−0.35	0.87	2.00	0.44	−0.14	0.68	1.29
Type of bystander-witness status	0.14	−0.37	1.09	3.25	0.25	0.46	1.13	2.97	0.37	−0.17	0.95	0.97
Bystander-initiated CPR	0.15	−0.63	1.004	1.38	0.22	−0.47	1.04	1.30	0.27	−0.11	1.14	0.996
Initially documented rhythms on the scene of cardiac arrest	−0.40	0.024	1.73	1.10	0.32	0.073	4.76	24.31	−0.72	−0.36	1.27	0.83
												
Pre-hospital procedures												
Advanced airway device	0.15	0.70	1.004	1.52	-0.77	0.066	1.27	1.04	-0.17	-0.11	1.09	1.04
Intravenous epinephrine administration	0.21	0.47	0.84	2.42	-0.95	-0.21	0.78	0.80	-0.33	-0.34	0.70	0.68
Defibrillation	1.65	0.0	0.98	1.0	1.27	1.27	8.26	8.00	0.52	0.26	2.47	1.38
ROSC until/on hospital arrival	0.46	-0.51	0.49	–	0.52	1.09	0.70	0.85	0.36	0.17	3.12	1.50
Emergency call to hospital arrival	-0.56	-0.36	0.12	0.097	-0.059	0.29	3.75	9.44	-0.45	-0.57	0.38	0.39
rSO_2_ at hospital arrival	-0.51	-0.051	0.52	1.72	0.21	0.071	0.70	1.37	0.39	0.28	1.43	1.26
Rhythms at rSO_2_ measurement	0.50	-0.45	0.39	–	0.34	1.03	1.02	0.93	-0.32	-0.20	2.16	1.50
												
Procedures after hospital arrival												
Coronary angiography	1.14	-0.19	1.47	1.22	0.98	1.10	4.23	7.93	0.94	0.99	5.45	6.77
Primary percutaneous coronary intervention	-0.098	-1.69	0.81	0.58	0.60	0.75	5.78	–	0.49	0.39	7.59	3.54

SD=standard deviation, CPR=cardiopulmonary resuscitation, ROSC=return of spontaneous circulation.

a SDs and variance ratios are results from estimating average treatment effects on the treated (ATT).

**Table 2:** Balance of covariates of propensity score matching analysis for favorable neurological outcomes^a^.

**Covariates**	**rSO**_**2**_**≥61%, G1 (*N* = 68, 34 pairs)**				**rSO**_**2**_**41–60%, G2 (*N* = 67, 31 pairs)**				**rSO**_**2**_**15–40%, G3 (N=296, 54 pairs)**			
	**SD**		**Variance ratio**		**SD**		**Variance ratio**		**SD**		**Variance ratio**	
	**Before matching**	**After matching**	**Before matching**	**After matching**	**Before matching**	**After matching**	**Before matching**	**After matching**	**Before matching**	**After matching**	**Before matching**	**After matching**
Sex	0.36	−0.37	0.65	2.63	0.25	1.09	0.82	1.00	0.28	0.040	0.86	0.97
Age	−0.38	−0.14	0.83	2.25	1.52	0.11	2.47	0.99	−0.18	−0.22	0.69	0.77
Location of cardiac arrest	0.37	−0.16	1.04	1.66	0.71	−0.40	1.59	0.78	0.041	−0.074	1.23	0.97
Witness	0.092	−0.58	0.84	–	0.13	−0.35	0.87	2.00	0.44	−0.14	0.68	1.29
Type of bystander-witness status	0.14	−0.37	1.09	3.25	0.25	0.46	1.13	2.97	0.37	−0.17	0.95	0.97
Bystander-initiated CPR	−0.15	−0.63	1.004	1.38	0.22	-0.47	1.04	1.30	0.27	−0.11	1.14	0.996
Initially documented rhythms on the scene of cardiac arrest	−0.40	0.024	1.73	1.10	0.32	0.073	4.76	24.31	−0.72	−0.36	1.27	0.83
												
Pre-hospital procedures												
Advanced airway devices	0.15	0.70	1.004	1.52	−0.77	0.066	1.27	1.04	−0.17	−0.11	1.09	1.04
Intravenous epinephrine administration	−0.21	0.47	0.84	2.42	−0.95	−0.21	0.78	0.80	−0.33	−0.34	0.70	0.68
Defibrillation	1.65	0.0	0.98	1.00	1.27	1.27	8.26	8.00	0.52	0.26	2.47	1.38
ROSC until/on hospital arrival	0.46	−0.51	0.49	–	0.52	1.09	0.70	0.85	0.36	0.17	3.11	1.50
Emergency call to hospital arrival	−0.56	−0.36	0.12	0.097	−0.059	0.29	3.75	9.44	−0.45	−0.57	0.38	0.39
rSO_2_ at hospital arrival	0.51	−0.051	0.52	1.72	0.21	0.071	0.70	1.37	0.39	0.28	1.43	1.26
Rhythms at rSO_2_ measurement	0.50	−0.45	0.39	–	0.34	1.03	1.02	0.93	−0.32	−0.20	2.16	1.50
												
Procedures after hospital arrival												
Coronary angiography	1.14	−0.19	1.47	1.22	0.98	1.10	4.23	7.93	0.94	0.99	5.45	6.77
Primary percutaneous coronary intervention	−0.098	−1.69	0.81	0.58	0.60	0.75	5.78	–	0.49	0.39	7.59	3.54

SD=standard deviation, CPR=cardiopulmonary resuscitation, ROSC=return of spontaneous circulation.

a SDs and variance ratios are results from estimating average treatment effects on the treated (ATT).

**Table 3:** Balance of covariates of inverse probability of treatment weighting for all-cause mortality^a^.

**Covariates**	**rSO2 ≥ 61%, G1 (*N* = 45)**				**rSO2 41–60%, G2 (*N* = 42)**				**rSO2 15–40%, G3 (*N* = 228)**			
	**SD**		**Variance ratio**		**SD**		**Variance ratio**		**SD**		**Variance ratio**	
	**Before weighted**	**After weighted**	**Before weighted**	**After weighted**	**Before weighted**	**After weighted**	**Before weighted**	**After weighted**	**Before weighted**	**After weighted**	**Before weighted**	**After weighted**
Sex	0.36	0.062	0.65	0.90	0.25	0.075	0.82	1.001	0.28	−0.061	0.86	1.02
Age	−0.38	0.069	0.83	1.06	−1.52	0.17	2.47	0.86	−0.18	−0.14	0.69	0.86
Location of cardiac arrest	0.37	−0.045	1.04	1.48	0.71	−0.29	1.59	0.75	0.41	0.21	1.23	1.14
Witness	0.092	−0.10	0.84	1.27	0.13	−0.54	0.87	1.50	0.44	0.13	0.68	0.92
Type of bystander-witness status	0.14	−0.17	1.09	1.43	0.25	−0.049	1.13	1.70	0.37	0.076	0.95	0.89
Bystander-initiated CPR	−0.15	−0.54	1.004	1.16	0.22	−0.47	1.04	0.94	0.27	0.080	1.14	1.04
Initially documented rhythms on the scene of cardiac arrest	−0.40	0.24	1.73	1.14	−0.32	0.17	4.76	4.43	−0.72	−0.44	1.27	0.79
												
Pre-hospital procedures												
Advanced airway devices	0.15	0.77	1.004	1.24	−0.77	−0.45	1.27	0.81	0.17	−0.060	1.09	1.03
Intravenous epinephrine administration	−0.21	0.21	0.84	1.31	−0.95	−0.59	0.78	0.58	0.33	−0.33	0.70	0.68
Defibrillation	1.65	0.44	0.98	0.82	1.27	0.76	8.26	6.93	0.52	0.26	2.47	1.62
ROSC at hospital arrival	0.46	0.093	0.49	0.85	0.52	0.35	0.70	1.09	0.36	0.00084	3.12	1.003
Emergency call to hospital arrival	0.56	−0.37	0.12	0.059	−0.059	0.00062	3.75	2.33	0.45	−0.43	0.38	0.36
rSO_2_ at hospital arrival	0.51	−0.38	0.52	0.69	0.21	−0.082	0.70	0.66	0.39	0.34	1.43	1.22
Rhythms at rSO_2_ measurement	0.50	0.20	0.39	0.54	0.34	0.61	1.02	0.70	−0.32	−0.39	2.16	1.21
												
Procedures after hospital arrival												
Coronary angiography	1.14	0.11	1.47	0.99	0.98	0.64	4.23	4.92	0.94	0.78	5.45	4.13
Primary percutaneous coronary intervention	−0.098	−1.02	0.81	0.27	0.60	0.42	5.78	6.73	0.49	0.44	7.59	5.01

SD = standard deviation, CPR = cardiopulmonary resuscitation, ROSC = return of spontaneous circulation.

a SDs and variance ratios are results from estimating average treatment effects (ATE).

**Table 4:** Balance of covariates of inverse probability of treatment weighting for favorable neurological outcomes^a^.

**Covariates**	**rSO2 ≥61%, G1 (*N* = 68)**				**rSO2 41–60%, G2 (*N* = 67)**				**rSO2 15–40%, G3 (*N* = 296)**			
	**SD**		**Variance ratio**		**SD**		**Variance ratio**		**SD**		**Variance ratio**	
	**Before weighted**	**After weighted**	**Before weighted**	**After weighted**	**Before weighted**	**After weighted**	**Before weighted**	**After weighted**	**Before weighted**	**After weighted**	**Before weighted**	**After weighted**
Sex	0.36	0.062	0.65	0.90	0.25	0.32	0.82	0.97	0.28	0.036	0.86	0.99
Age	−0.38	0.069	0.83	1.06	1.52	−0.0051	2.47	0.77	−0.18	−0.28	0.69	0.63
Location of cardiac arrest	0.37	−0.045	1.04	1.48	0.71	0.092	1.59	0.76	0.41	0.11	1.23	1.21
Witness	0.092	−0.10	0.84	1.27	0.13	−0.22	0.87	1.32	0.44	0.022	0.68	0.99
Type of bystander-witness status	0.14	−0.17	1.09	1.43	0.25	0.17	1.13	1.49	0.37	-0.074	0.95	0.77
Bystander-initiated CPR	−0.15	−0.54	1.004	1.16	0.22	−0.32	1.04	0.99	0.27	0.0072	1.14	1.004
Initially documented rhythms on the scene of cardiac arrest	0.40	0.24	1.73	1.14	−0.32	0.25	4.76	4.85	−0.72	−0.75	1.27	0.59
												
Pre-hospital procedures												
Advanced airway devices	0.15	0.77	1.004	1.24	−0.77	−0.35	1.27	0.96	−0.17	−0.43	1.09	1.05
Intravenous epinephrine administration	−0.21	0.21	0.84	1.31	−0.95	−0.76	0.78	0.53	−0.33	−0.37	0.70	0.64
Defibrillation	1.65	0.44	0.98	0.82	1.27	0.77	8.26	6.85	0.52	0.064	2.47	1.16
ROSC at hospital arrival	0.46	0.093	0.49	0.85	0.52	0.16	0.70	1.03	0.36	0.059	3.12	1.25
Emergency call to hospital arrival	0.56	−0.37	0.12	0.059	0.059	−0.082	3.73	2.67	0.45	−0.28	0.38	0.44
rSO_2_ at hospital arrival	−0.51	−0.38	0.52	0.69	0.21	0.00	0.70	0.81	0.39	0.086	1.43	1.10
Rhythms at rSO_2_ measurement	0.50	0.20	0.39	0.54	0.34	0.18	1.02	1.04	0.32	−0.26	2.16	1.23
												
Procedures after hospital arrival												
Coronary angiography	1.14	0.11	1.47	0.99	0.98	0.56	4.23	4.00	0.94	0.51	5.45	3.87
Primary percutaneous coronary intervention	−0.098	−1.02	0.81	0.27	0.60	0.40	5.78	6.32	0.49	0.21	7.59	2.87

SD=standard deviation, CPR=cardiopulmonary resuscitation, ROSC=return of spontaneous circulation.

a SDs and variance ratios are results from estimating average treatment effects (ATE).

**Table 5:** Analysis results on the effectiveness of target temperature management (32–34 °C) for all-cause mortality or favorable neurological outcomes of patients those who achieved return of spontaneous circulation until/on hospital arrival (*n* = 117).

	**Effectiveness of TTM (32–34℃) on all-cause mortality**			**Effectiveness of TTM (32–34℃) on favorable outcomes (CPC 1–2)**		
	**rSO**_**2**_**≥61%, G1 (n=54)**	**rSO**_**2**_**41–60%, G2 (n=43)**	**rSO**_**2**_**15–40%, G3 (n=20)**	**rSO**_**2**_**≥61%, G1 (n=54)**	**rSO**_**2**_**41–60%, G2 (n=43)**	**rSO**_**2**_**15–40%, G3 (n=20)**
**Univariate analysis**						
**Risk ratio**	0.29	0.36	0.70	1.87	11.52	1.22
**[95%CI]**	[0.11 to 0.80]	[0.20 to 0.65]	[0.30 to 1.64]	[1.06 to 3.29]	[1.68 to 79.15]	[0.32 to 4.65]
**Risk difference**	0.33	-0.57	-0.19	0.33	0.58	0.061
**[95%CI]**	[-0.56 to -0.091]	[-0.80 to -0.34]	[-0.62 to 0.24]	[0.071 to 0.58]	[0.37 to 0.80]	[-0.34 to 0.47]
						
**Multivariate logistic regression**^a^						
**Odds ratio**	0.36	0.16	4.65e-06	1.33	22.63	1.25
**[95%CI]**	[0.040 to 3.25]	[0.0061 to 4.33]	[5.11e-14 to 423.43]	[0.25 to 7.11]	[0.50 to 1016.29]	[0.13 to 12.47]
						
**Propensity-score matching**^b^						
**ATE**	-0.074	-0.63	-0.15	0.074	0.63	0.050
**[95%CI]**	[-0.42 to 0.27]	[-0.86 to -0.40]	[-0.66 to 0.36]	[-0.012 to 0.16]	[0.40 to 0.86]	[-0.22 to 0.32]
**ATT**	0.033	-0.68	-0.44	-0.067	0.64	0.22
**[95%CI]**	[-0.17 to 0.24]	[-0.86 to -0.50]	[-0.74 to -0.14]	[-0.33 to 0.20]	[0.46 to 0.82]	[-0.19 to 0.64]
						
**IPW**^b^						
**ATE**	-0.051	-0.52	-0.29	0.061	0.53	0.045
**[95%CI]**	[-0.30 to 0.19]	[-0.78 to -0.26]	[-0.55 to -0.038]	[-0.19 to 0.31]	[0.28 to 0.78]	[-0.29 to 0.38]
**ATT**	0.034	-0.64	-0.42	-0.098	0.61	0.22
**[95%CI]**	[-0.18 to 0.25]	[-0.84 to -0.44]	[-0.72 to -0.12]	[-0.37 to 0.18]	[0.40 to 0.81]	[-0.18 to 0.62]

TTM=target temperature management, CPC=cerebral performance category, ATE=average treatment effect, ATT=average treatment effect on the treated, IPW=inverse probability of treatment weighting.

a In multivariate logistic analysis, explanatory variables including sex, age, witnessed arrest, PaO2, PaCO2, first monitored rhythm (shockable [VF/pulseless VT]/non-shockable [PEA, asystole, unknown]) were used for statistical adjustment.

b We used age, sex, witnessed arrest, PaO2, PaCO2, first monitored rhythm (shockable [VF/pulseless VT] / non-shockable [PEA, asystole, unknown]) as covariates for estimating the PS, and if possible, more variables relating to patient characteristics observed before TTM were also used.

## 2 Experimental design, materials, and methods

### 2.1 Study design and data source

The original research article is a secondary analysis of prospectively collected registry, the Japan-Prediction of Neurological Outcomes in Patients Post-cardiac Arrest Registry [UMIN trial ID 000005065] [2], [3], in which OHCA patients transported to 15 tertiary emergency hospitals in Japan from May 2011 to August 2013 were consecutively registered. The database consists of pre-hospital and in-hospital data collected from the Japanese emergency medical service (EMS) system and medical charts of each hospital by using the Utstein style [4].

### 2.2 Study population

Comatose patients after OHCA were included in this study if they achieved ROSC. Exclusion criteria were trauma, accidental hypothermia, age <18 years, completion of “Do Not Resuscitate [5]” orders, and a Glasgow coma scale (GCS) score of >8 on arrival at the hospital.

After arriving at hospital, two disposable probes of NIRS (INVOS TM 5100C, Covidien, Boulder, CO, USA) were attached to the patient's forehead. rSO_2_ was monitored at least for 1 minute with the probes after several seconds of stable monitoring, and the lowest rSO_2_ value was used.

Patients were stratified into three groups according to the recorded rSO_2_: group rSO_2_ ≥61% (G1), group rSO_2_ 41–60% (G2), and group rSO_2_ ≤40% (G3), by referring to previous studies which suggest that values less than 35–40% or an absolute decrease of 20% from baseline should alert clinicians to perform appropriate interventions to reverse potential cerebral hypoxemia [6], [7], [8], [9], [10], and reported that rSO_2_ values are 60% or higher in most stable patients [7], [9], [11].

### 2.3 Variables

#### 2.3.1 Treatment and outcome measurement

The treatment, TTM with 32 to 34°C (12–24 h) was conducted by the discretion of the attending physician.

We defined the primary outcome as all-cause mortality at 90 days after cardiac arrest, and the secondary outcome as favorable neurological outcome evaluated according to the Cerebral Performance Category (CPC) [12]. The CPC is a 5-point scale ranging from 1 (good cerebral performance) to 5 (dead). We defined favorable neurological outcome as a CPC 1 or 2 by reference to the international guidelines [13], [14]. Both all-cause mortality and neurological outcome are core elements in the guidelines. In principle, CPC in individual patients were determined by the physician-in-charge, but in cases of missing data, the main researcher who developed the database determined CPC by contacting patients or family members; both were blinded to rSO_2_ readings.

#### 2.3.2 Covariates

We used patient characteristics as covariates, including demographic characteristics (sex, age), pre-hospital status (location of arrest, witnessed arrest, bystander CPR, first monitored rhythm), pre-hospital resuscitation attempts by EMS (airway management by intubation or laryngeal mask airway device, intravenous injection of adrenaline, usage of Automated External Defibrillator [AED]), patient status at emergency unit (time from emergency call to hospital arrival, rhythm of electrocardiogram on rSO_2_ measurement), cardiac origin or not (presumed by attending physician retrospectively), and procedures after hospitalization (ECPR, coronary angiography, primary percutaneous coronary intervention).

### 2.4 Statistical analyses

In original research article, effectiveness of TTM was evaluated by group according to rSO_2_. Risk ratios and risk differences were obtained by univariate analyses. In multivariate logistic analysis, explanatory variables including sex, age, witnessed arrest, PaO2, PaCO2, first monitored rhythm (shockable [VF/pulseless VT] / non-shockable [PEA, asystole, unknown]) were used for statistical adjustment. Treatment effect estimation was also performed by propensity-score matching (PSM) and inverse-probability weighting (IPW), in order to adjust for differences in baseline characteristics [15], [16], [17], [18]. All analyses were performed with Stata SE, version 14.0 (Stata Corp., College Station, TX, USA). Tests of statistical significance were two-tailed with an alpha of 0.05.

Potential-outcome models, also known as Rubin causal models, were used to estimate the distribution of individual-level treatment effects, i.e., changes in outcome caused by receiving one treatment over another [17], [18]. We used the average treatment effect (ATE: average effect of the treatment in the population) and average treatment effect on the treated (ATT: average treatment effect among those who received the treatment).

In PSM analysis, we performed nearest neighbor matching within caliper [16]. We basically used age, sex, witnessed arrest, PaO2, PaCO2 and first monitored rhythm (shockable / non-shockable) as covariates for estimating the propensity score (PS), and if possible, more variables relating to patient characteristics observed before TTM were also used to increase the accuracy of the PS model. We used calipers of width 0.2*(SD of log PS) for matching and also included interaction and higher order terms. In IPW analysis, we basically used same covariates as PSM, and if possible, more variables observed before TTM were used, including interaction and higher order terms. We showed balances of covariates (Table 1, Table 2, Table 3, Table 4) and overlap plots (Fig. 1, Fig. 2) of PSM and IPW analysis. Sensitivity analyses were performed by limiting patients to those who achieved ROSC upon hospitals arrival (excluding patients with ROSC after arrival) (Table 5).

## Footnotes

Appendix A Supplementary data associated with this article can be found in the online version at 10.1016/j.dib.2018.02.050.
